# Iron oxide nanoparticles induce reversible endothelial-to-mesenchymal transition in vascular endothelial cells at acutely non-cytotoxic concentrations

**DOI:** 10.1186/s12989-019-0314-4

**Published:** 2019-07-12

**Authors:** Tao Wen, Lifan Du, Bo Chen, Doudou Yan, Aiyun Yang, Jian Liu, Ning Gu, Jie Meng, Haiyan Xu

**Affiliations:** 1 Institute of Basic Medical Sciences, Chinese Academy of Medical Sciences & Peking Union Medical College, Beijing, 100005 China; 2 Materials Science and Devices Institute, Suzhou University of science and Technology, Suzhou, 215009 China; 3 State Key Laboratory of Bioelectronics, Jiangsu Key Laboratory for Biomaterials and Devices, School of Biological Science and Medical Engineering, Southeast University, Nanjing, 210096 China

**Keywords:** Iron oxide nanoparticle, Endothelial-to-mesenchymal transition, Reactive oxygen species, Reversible

## Abstract

**Background:**

Iron oxide nanoparticles (IONPs) have been extensively studied in different biomedical fields. Recently, the non-cytotoxic concentration of IONPs induced cell-specific response raised concern of their safety. Endothelial cell exposure was unavoidable in their applications, while whether IONPs affect the phenotype of vascular endothelial cells is largely unknown. In this work, the effect of IONPs on endothelial-to-mesenchymal transition (EndMT) was investigated in vitro and in vivo.

**Results:**

The incubation with γ-Fe_2_O_3_ nanoparticles modified with polyglucose sorbitol carboxymethyether (PSC-Fe_2_O_3_) at non-cytotoxic concentration induced morphological changes of human umbilical vein endothelial cells (HUVECs) from cobblestone-like to spindle mesenchymal-like morphology, while PSC-Fe_2_O_3_ mostly stay in the culture medium and intercellular space. At the same time, the endothelial marker CD31 and VE-cadherin was decreased along with the inhibitory of angiogenesis properties of HUVEC. Meanwhile, the mesenchymal marker α-smooth muscle actin (α-SMA) and fibroblast specific protein (FSP) was up regulated significantly, and the migration ability of the cells was enhanced. When ROS scavenger mannitol or AA was supplemented, the EndMT was rescued. Results from the in vivo study showed that, expression of CD31 was decreased and α-SMA increased in the liver, spleen and kidney of mice given PSC-Fe_2_O_3_, and the density of collagen fibers in the liver sinusoid of mice was increased. The supplementary mannitol or AA could reverse the degree of EndMT in the tissues. Mechanistic study in vitro indicated that the level of extracellular hydroxyl radicals (·OH) was up regulated significantly by PSC-Fe_2_O_3_, which induced the response of intracellular ROS and resulted in the EndMT effect on HUVECs.

**Conclusion:**

The PSC-Fe_2_O_3_ was capable of inducing EndMT in the endothelial cells at acutely non-cytotoxic dose due to its intrinsic peroxidase-like activity, though they were few taken up by endothelial cell. The EndMT effect on HUVEC can be rescued by ROS scavenger in vitro and in vivo.

**Electronic supplementary material:**

The online version of this article (10.1186/s12989-019-0314-4) contains supplementary material, which is available to authorized users.

## Background

Iron oxide nanoparticles (IONPs) have been intensively investigated and developed in many biomedical fields, such as intravenous cell targeting, labeling and separation, magnetic resonance imaging contrast, drug or gene delivery system, and hyperthermia [1–3], because they show a low cytotoxicity in a quite large range of tested concentration [4, 5]. Nevertheless, potential effects of IONPs at non-cytotoxic concentrations on human health have been explored in recent years, especially on vascular endothelial cells, because blood vessel is one of the major barriers for INOPs intended to use as therapeutics and diagnostics. For examples, it was reported that IONPs exposure induced the elongated morphology and increased elastic modulus of endothelial cells [6, 7]. Even no cytotoxicity was detected, at low amount of IONPs, the endocrine and urea transporter function, inflammatory responses, angiogenesis function of endothelial cells (ECs) were found to change [8]. The endothelial integrity was also reported to alter via interplay with barrier function and attenuation of cytoprotective and anti-inflammatory NO production at non-cytotoxic concentration of IONPs [9]. These cues imply possible effects of IONPs at non-cytotoxic concentration on the phenotype of ECs.

Endothelium to mesenchymal transition (EndMT) is one changed phenotype of endothelial cells that represents losing endothelial characteristics and gaining a mesenchymal phenotype [10], which have a significant role in some diseases, especially fibrosis in liver [11, 12], kidney [13], cardiac [14], and atherosclerosis [15], diabetes [16] and cancer [17]. It also has been documented that endothelial cells can undergo EndMT in certain physiological environmental stimulations including growth factor TGFβ [18], materials stiffness [19], and stretching stress [20]. Whether IONPs can induce EndMT is unknown and worthy to note.

In this work, whether γ-Fe_2_O_3_ nanoparticles modified with polyglucose sorbitol carboxymethyether (PSC-Fe_2_O_3_) were able to inducing EndMT upon human umbilical vein endothelial cells (HUVECs) was investigated in vitro and in vivo. We showed that PSC-Fe_2_O_3_ at acutely non-cytotoxic concentration significantly reduced the expression of endothelial marker CD31 and VE-cadherin, meanwhile increased the mesenchymal marker α-smooth muscle actin (α-SMA) and fibroblast specific protein (FSP) due to the peroxidase-like activity, by which the migration of endothelial cells was enhanced and their angiogenic function was inhibited, clearly indicating the occurrence of EndMT in the endothelial cells. The underlying mechanism of PSC-Fe_2_O_3_ induced EndMT was also elucidated.

## Results

### Characterization and cytotoxicity of PSC-Fe_2_O_3_

**Fig. 1:**
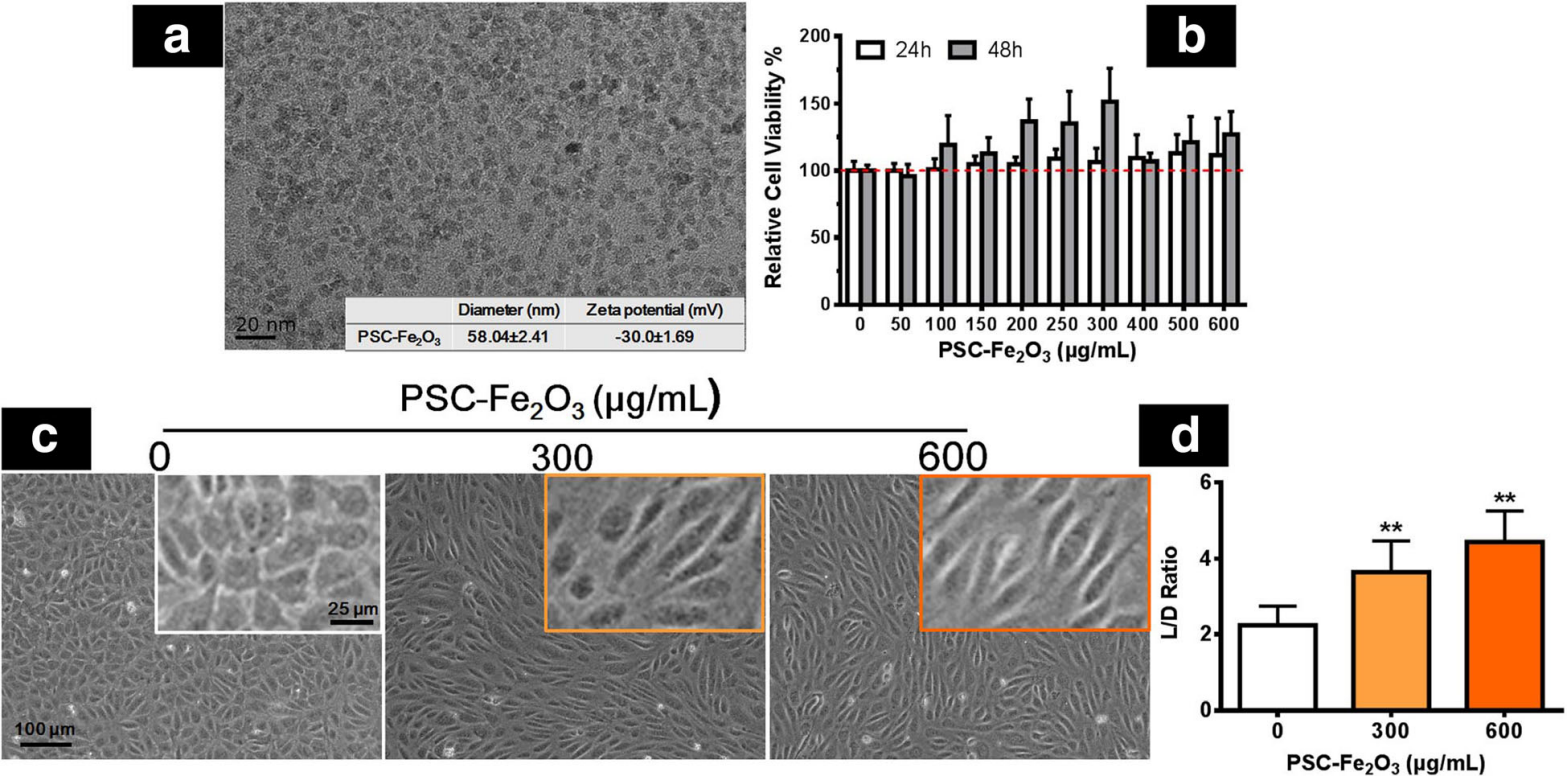
**a** TEM image of PSC-Fe_2_O_3_, inserted provided the average dynamic diameter and Zeta potential of PSC-Fe_2_O_3_ in water. **b** Cell viability of HUVECs treated with PSC-Fe_2_O_3_ at different concentrations (*n* = 3). **c** Representative morphology of HUVECs treated with PSC-Fe_2_O_3_ at 300 and 600 μg/mL. **d** The average L/D ratio of HUVECs treated with different concentration of PSC-Fe_2_O_3_ (*n* = 30). Data are mean ± SD, one-way ANOVA with Dunnett’s multiple comparisons test, ** *p* < 0.01, significant compared to control

The crystal core of PSC-Fe_2_O_3_ was about 10 nm in diameter (Fig. 1a), and the average hydrodynamic diameter was 58 nm with − 30 mV of zeta potential. It should be noted that PSC-Fe_2_O_3_ did not show any cytotoxicity to HUVECs when its concentration was reached at 600 μg/mL (Fig. 1b), however, the morphology of the cells changed significantly, showing a spindle mesenchymal-like morphology at the concentration while losing their endothelial characteristic polygonal shape (Fig. 1c). The ratio of length to diameter (L/D) for the endothelial cells was two folds of that of control group when incubated with 600 μg/mL PSC-Fe_2_O_3_ (Fig. 1d). When observed at a lower confluence of 70% after PSC-Fe_2_O_3_ treatment, HUVECs also became spindle mesenchymal-like morphology (Additional file 1: Figure S1).

### PSC-Fe_2_O_3_ induce EndMT

**Fig. 2:**
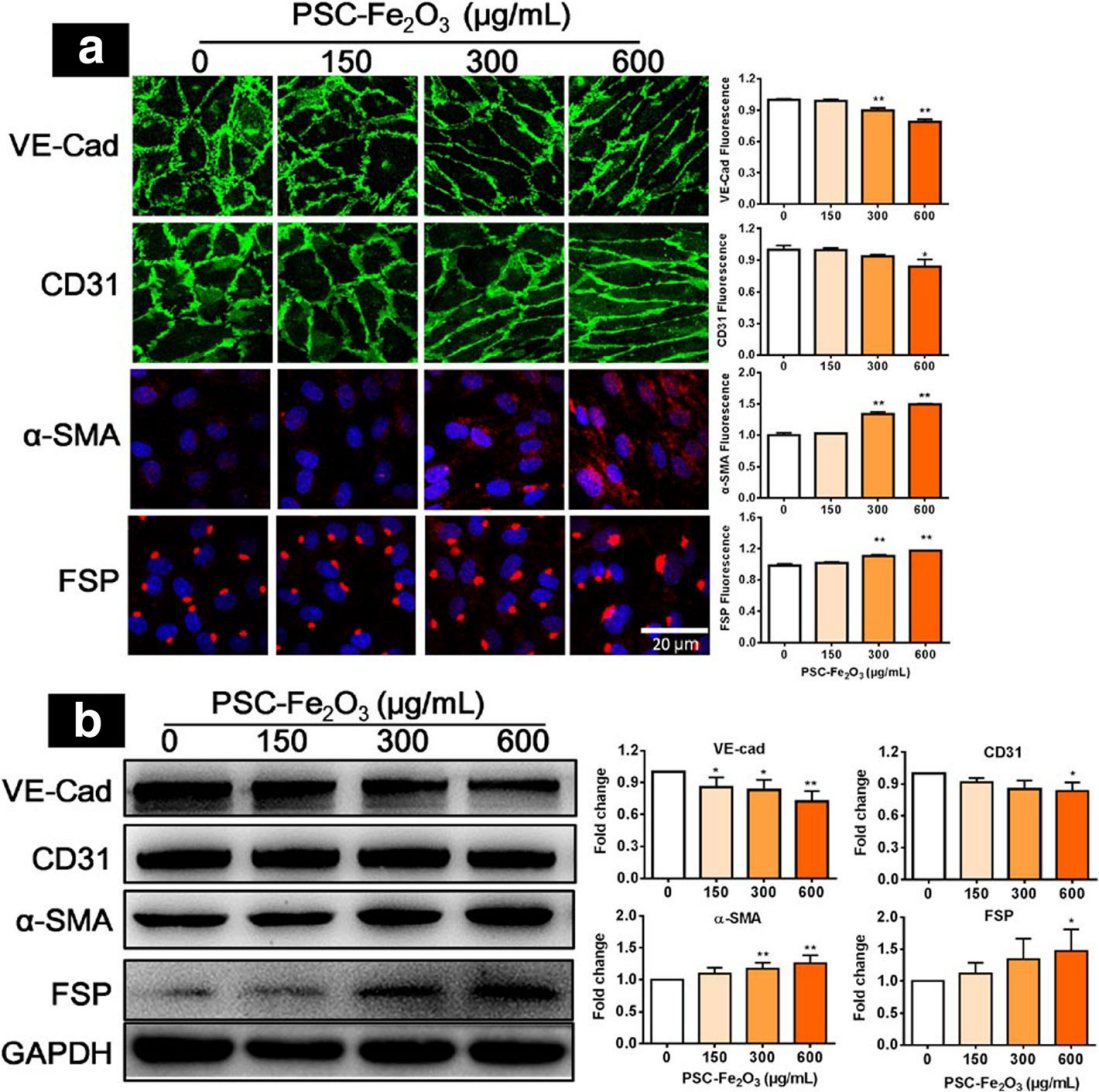
PSC-Fe_2_O_3_ at non-cytotoxic concentration induced EndMT in HUVECs. **a** Representative fluorescence confocal microphotographs and quantitative fluorescence intensity of HUVECs immunostatined with endothelial cell marker CD31 (green) or VE-cadherin (green) and mesenchymal marker α-SMA (red) or FSP (red), counterstained with DAPI for nuclei (blue). **b** Representative immunoblots and quantification of CD31, VE-cadherin, α-SMA, FSP of protein lysates from HUVECs treated with PSC-Fe_2_O_3_. Data are mean ± SD, n = 3, one-way ANOVA with Dunnett’s multiple comparisons test, *p < 0.05, ** *p* < 0.01, significant compared to control

Next we investigated what happened on the phenotype of HUVECs when their morphology changed largely. The expression of CD31 and VE-cadherin (VE-cad) was examined by immunoblotting and immunofluorescence upon HUVECs incubated with PSC-Fe_2_O_3_ at different concentrations. It was shown that the level of VE-cad was down regulated when PSC-Fe_2_O_3_ was at 300 μg/mL or 600 μg/mL, and CD31 was decreased as well when PSC-Fe_2_O_3_ was reached 600 μg/mL, while the expression of α-SMA and FSP significantly increased in the tested concentrations (Fig. 2a, b). These results clearly indicated that PSC-Fe_2_O_3_ was capable of inducing endothelial cell transformation to mesenchymal cells (EndMT) process at the acutely non-cytotoxic concentration.

### PSC-Fe_2_O_3_ mainly stayed in the culture medium and intercellular space

**Fig. 3:**
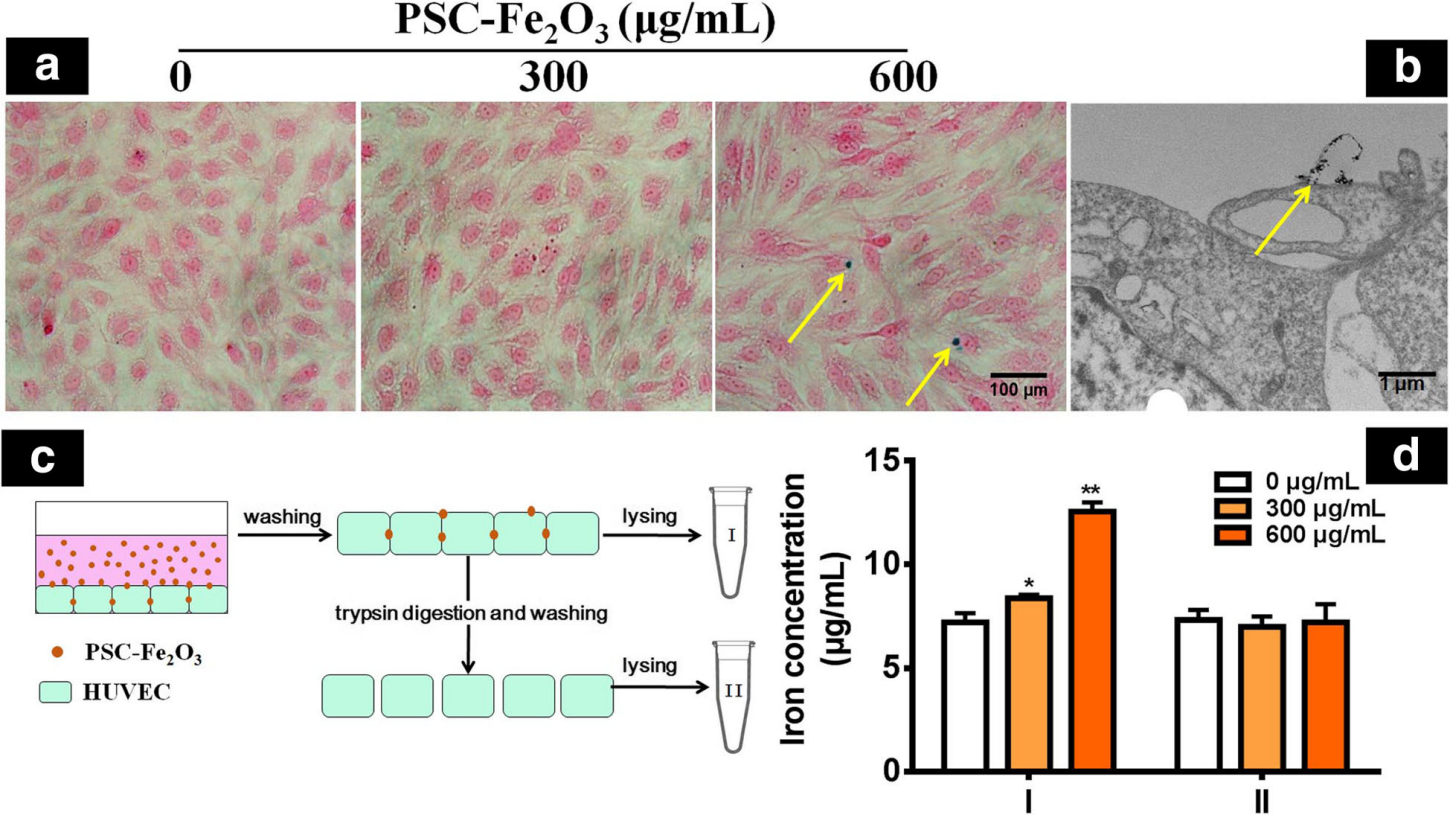
PSC-Fe_2_O_3_ located in the intercellular space rather not engulfed by HUVECs. **a** Photograph from microscope observation of cells with Prussian blue staining. **b** TEM images of HUVECs incubated with PSC-Fe_2_O_3_. **c** Scheme of procedures for preparing lysates and iron quantification. **d** Content of iron in the cell lysates prepared by procedure I or II. Data are mean ± SD, n = 3, one-way ANOVA with Dunnett’s multiple comparisons test, *p < 0.05, ** p < 0.01, significant compared to control

To understand how EndMT was triggered by PSC-Fe_2_O_3_, we first examined whether PSC-Fe_2_O_3_ were internalized by HUVECs. Results from Prussian blue Staining showed little blue color was observed in the cytoplasm of HUVEC incubated with PSC-Fe_2_O_3_, only sparkle of blue staining was seen between the cells when PSC-Fe_2_O_3_ was at 600 μg/mL (Fig. 3a), suggesting few PSC-Fe_2_O_3_ were internalized. With TEM, no appreciable PSC-Fe_2_O_3_ was observed inside the cells; instead, a small part of PSC-Fe_2_O_3_ located in the intercellular space of the cells (Fig. 3b), and most of PSC-Fe_2_O_3_ (more than 99%) stayed in the culture medium, which was determined by phenanthroline spectrophotometric method. To further validate above observations, cell lysates were prepared by two procedures and the iron content of the lysates was determined by phenanthroline method (Fig. 3c). It was shown that as for the samples prepared by lysis only (procedure I), the iron content of PSC-Fe_2_O_3_ groups was significantly higher than that of control group and concentration dependent. While for the samples prepared by the lysis following trypsin digestion (procedure II), no difference of iron content was observed between the control group and the two PSC-Fe_2_O_3_ groups (Fig. 3d). Because the trypsin digestion made the cells disconnected, therefore the increased iron content in lysates of procedure I was attributable to the PSC-Fe_2_O_3_ locating in the intercellular space. At the same time, the results provided further evidence of few PSC-Fe_2_O_3_ were taken up.

### Extracellularly PSC-Fe_2_O_3_ induced ROS increase by peroxidase-like activities

**Fig. 4:**
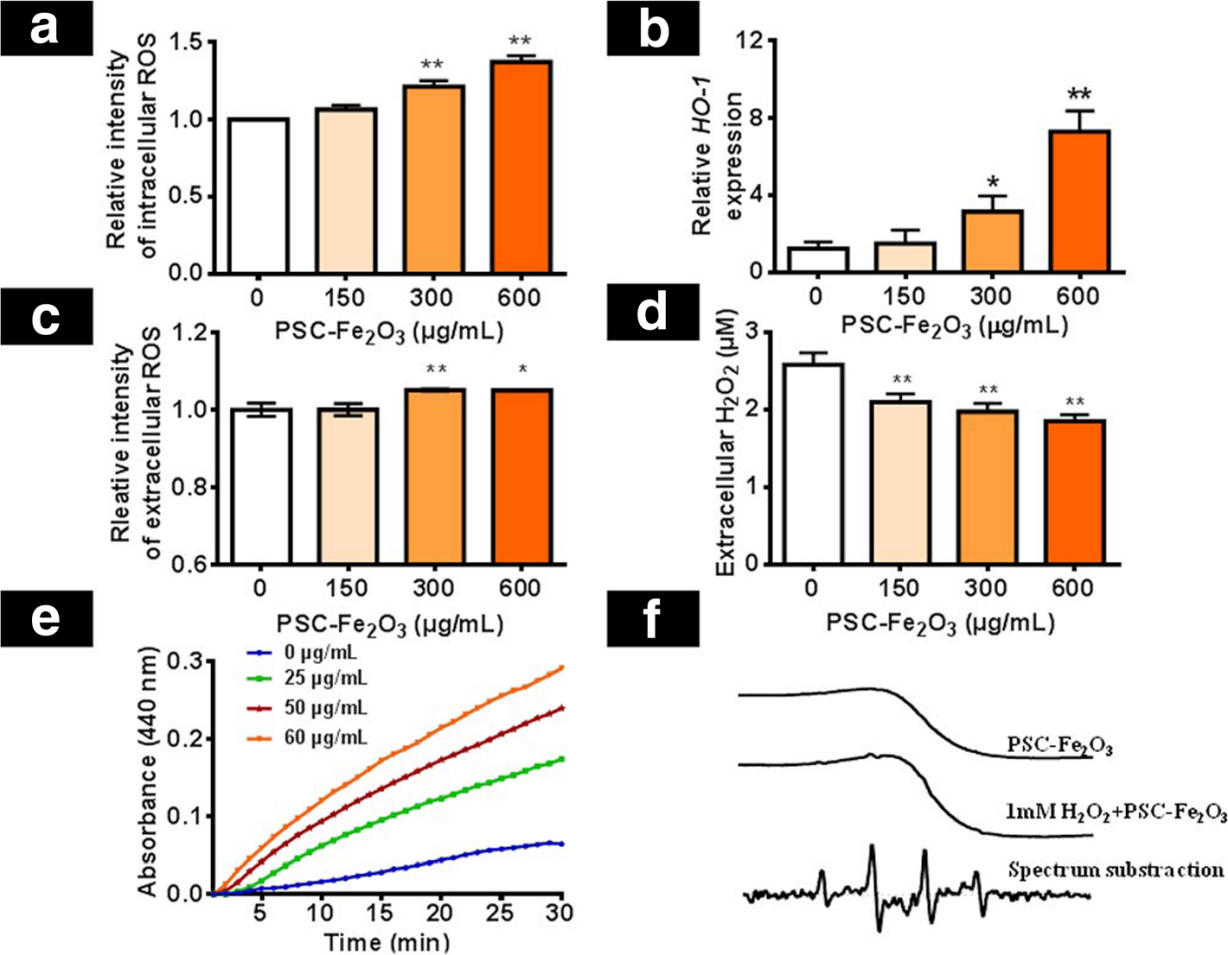
PSC-Fe_2_O_3_ induced ·OH production by peroxidase-like activities. **a** Intracellular ROS of HUVEC. **b** HO-1 gene expression of HUVEC. **c** Extracellular ROS of HUVEC. **d** Extracellular H_2_O_2_. **e** PSC-Fe_2_O_3_ catalyzed H_2_O_2_ into ·OH in a concentration dependent manner. **f** The ESR spectrum of DMPO / ·OH adduct. Data are mean ± SD, n = 3, one-way ANOVA with Dunnett’s multiple comparisons test, *p < 0.05, **p < 0.01, significant compared to control

Internalized nanoparticles usually up-regulate intracellular ROS [21, 22]. Although PSC-Fe_2_O_3_ mainly located in the culture medium and intercellular space instead of being taken up by endothelial cells, the impact of PSC-Fe_2_O_3_ on the intracellular ROS was also examined. It was striking that the intracellular ROS of HUVECs was increased when incubated with PSC-Fe_2_O_3_, and the effect was in concentration dependent manner (Fig. 4a). We also examined the change of ROS stress marker *HO-1* at gene level. Results showed that the expression of *HO-1* was increased in dose-dependent manner when HUVECs were exposed with PSC-Fe_2_O_3_ (Fig. 4b), which confirmed that ROS played a key role in PSC-Fe_2_O_3_-induced EndMT. At the same time, the extracellular ROS was increased and H_2_O_2_ content decreased (Fig. 4b, c), which was concentration dependent too. It has been documented that IONPs is capable of decomposing H_2_O_2_ into hydroxyl radicals (·OH) due to its peroxidase-like activity [23], we next accessed the peroxidase-like activity of PSC-Fe_2_O_3_ upon the commonly used peroxidase colorimetric substrates o-phenylenediamine (OPD) in the presence of H_2_O_2_ [24]. As Fig. 4d proved, PSC-Fe_2_O_3_ catalyzed H_2_O_2_ into ·OH in a concentration dependent manner, showing the peroxidase property. Furthermore, we detected ·OH directly by electron spin resonance (ESR) spectroscopy with the help of a spin trap 5, 5-Dimethyl-1-pyrroline N-oxide (DMPO). The ESR spectrum of the spin adduct DMPO/·OH showed four lines with relative intensities of 1:2:2:1 and hyperfine splitting parameters of a_N_ = 13.56, ${\mathrm{a}}_{\mathrm{H}}^{\upbeta}$ = 12.30, and ${\mathrm{a}}_{\mathrm{H}}^{\upgamma}$ = 0.66 [25] (Fig. 4f), which provided evidence that there was increased ·OH in the extracellular environment. Taken together above, we demonstrated that extracellular PSC-Fe_2_O_3_ decomposed H_2_O_2_, which resulted in the production of extracellular ·OH that induced up-regulation of intracellular ROS.

### ROS scavengers reverse EndMT of HUVEC

**Fig. 5:**
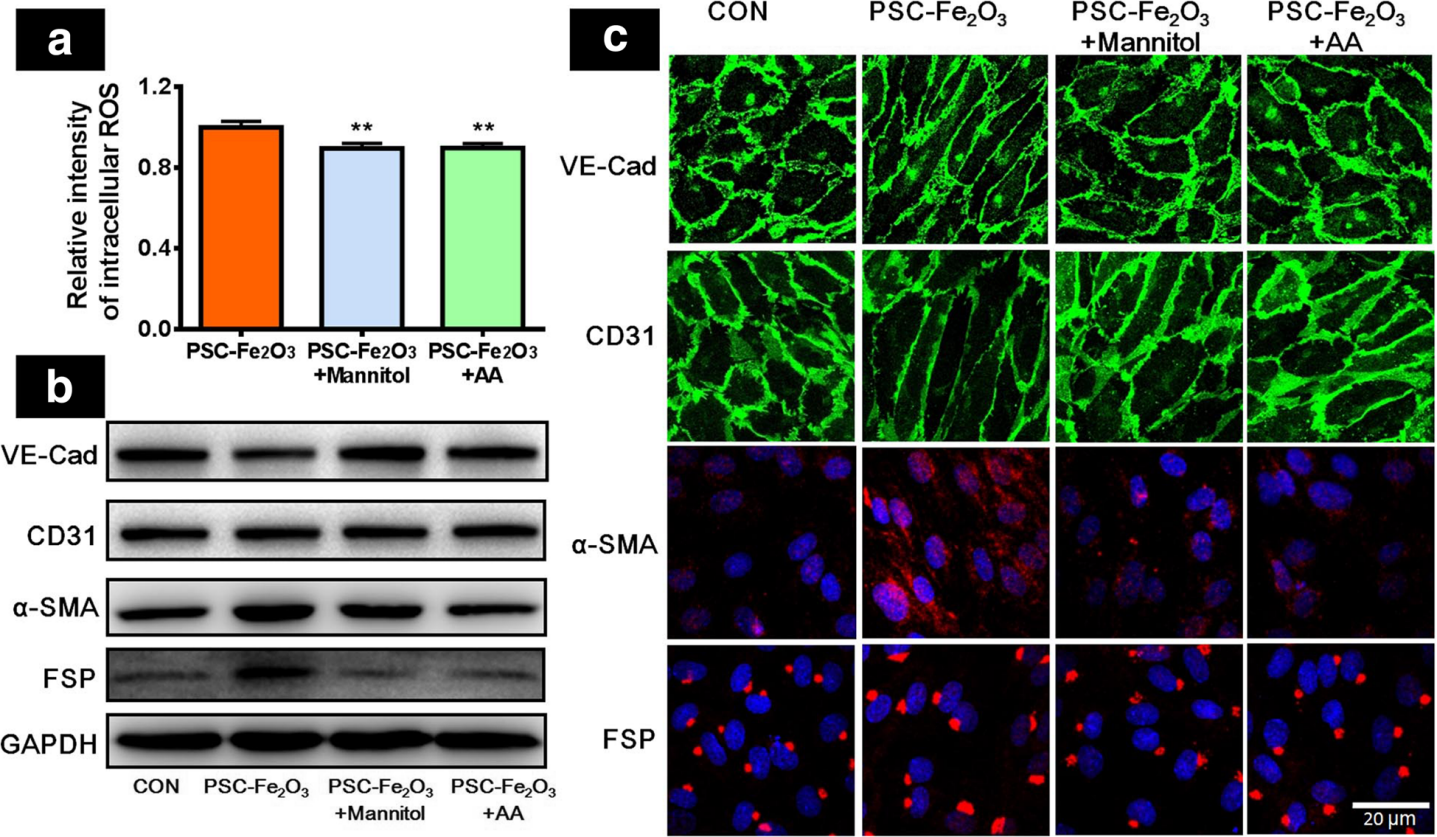
ROS scavengers rescued the EndMT induced by PSC-Fe_2_O_3_. **a** Intracellular ROS of PSC-Fe_2_O_3_ treated HUVECs in the absence and presence of mannitol or AA. **b** The level of VE-cad, CD31, α-SMA and FSP determined by western blot. **c** Expression of VE-cad (green), CD31 (green), α-SMA (red) and FSP (red) for HUVECs by confocal microscopy. Data are mean ± SD, n = 3, one-way ANOVA with Fisher’s least significant difference test (LSD-t), ** *p* < 0.01, significant compared to control

Some reports suggested that the up-regulation of intracellular ROS was one crucial factor inducing EndMT [26, 27], therefore we next investigated the relationship between the EndMT and IONP-induced ROS. It was shown that the supplement of hydroxyl radicals (·OH) scavenger mannitol [28] or non-specific ROS scavenger ascorbic acid (AA) [29] was capable of reducing the intracellular ROS level of HUVECs incubated with PSC-Fe_2_O_3_ (Fig. 5a). Moreover, either mannitol or AA was able to increase the expression of VE-Cad and CD31 while to decrease α-SMA and FSP for HUVEC in the presence of PSC-Fe_2_O_3_ (Fig. 5b and c). Although the change was not large at each immunoblots, the CD31 was decreased by PSC-Fe_2_O_3_ at 600 μg/mL with statistical significance (Additional file 1: Figure S2A and B). To confirm this in further, we conducted additional experiments to investigate the change of CD31 from gene level. It was shown that CD31 was down regulated in the present of 600 μg/mL PSC-Fe_2_O_3_ and could be rescued by adding AA (Additional file 1: Figure S2C). These results strongly suggested that PSC-Fe_2_O_3_ induced extracellular ·OH was one crucial factor for the occurrence of EndMT, which could be scavenged by the supplement of antioxidants.

**Fig. 6:**
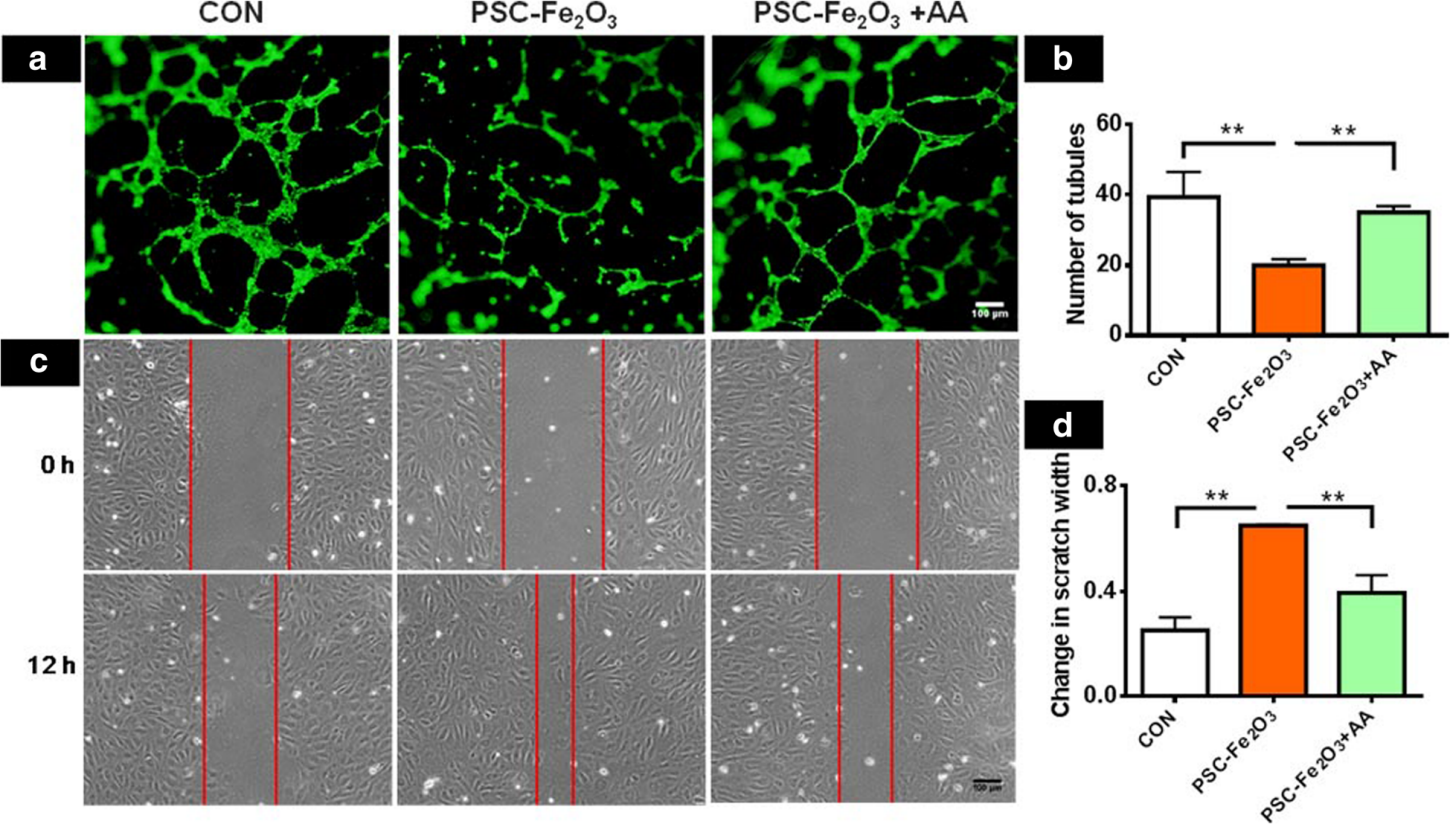
ROS scavenger AA reversed the function of endothelial cells occurring EndMT. **a** AA reversed tubule formation impairment of endothelial cell caused by PSC-Fe_2_O_3_. **b** Quantification of endothelial meshes (*n* = 3). **c** Images of scratch morphology changing with time. **d** Relative scratch width change (*n* = 2). Data are mean ± SD, one-way ANOVA with LSD-t, ** *p* < 0.01. Bar is 100 μm for all images

As EndMT can inhibit the function of angiogenesis and promote invasion for endothelial cells [30, 31], we examined the ability of HUVECs to form vessel-like structures in the presence of PSC-Fe_2_O_3_. The observation under microscope showed that the tubules formed by control HUVECs were dense, complete and continuous, while those formed by HUVECs incubated with PSC-Fe_2_O_3_ were less, only sparse network and few branches were observed. When supplemented with AA, the HUVECs incubated with PSC-Fe_2_O_3_ recovered their angiogenic ability, evidenced by the increased tubule formation (Fig. 6a). Quantification results showed that the number of meshes was significantly different between control group and PSC-Fe_2_O_3_ group, and between PSC-Fe_2_O_3_ group and PSC-Fe_2_O_3_ with AA group (Fig. 6b). At the same time, the covering rate of wound scratch was faster for PSC-Fe_2_O_3_ group than that for the control group, indicating the function of migration and invasion of the cells was enhanced, and which could also be reverted by adding AA (Fig. 6c, d).

### ROS scavengers rescued PSC-Fe_2_O_3_ induced EndMT in vivo

**Fig. 7:**
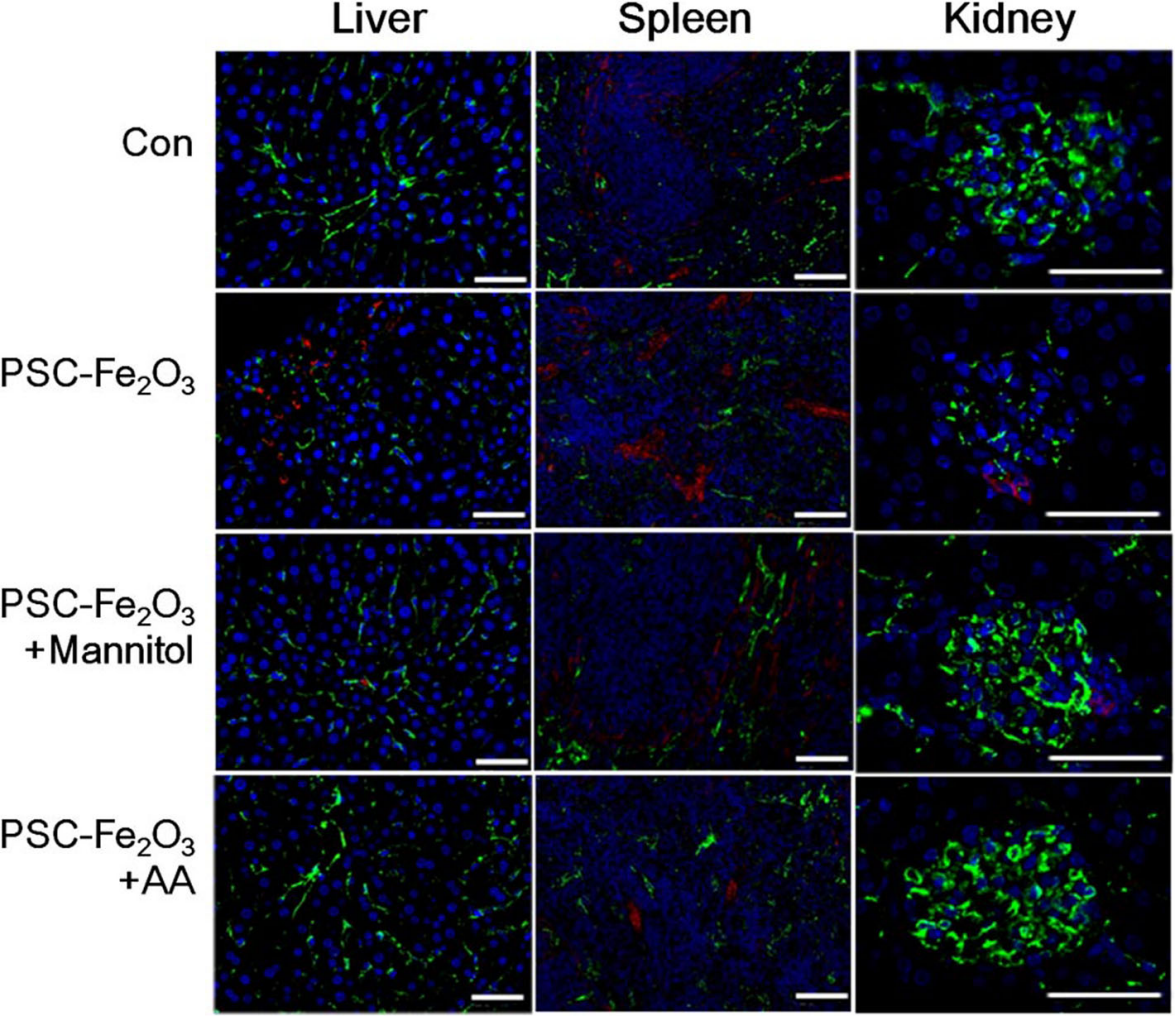
Representative of liver, spleen and kidney sections stained for mesenchymal markers α-SMA (red), the endothelial markers CD31 (Green), and nuclei (blue). Bar is 50 μm for all images

The EndMT induced by PSC-Fe_2_O_3_ was further investigated in healthy Balb/c mice. The mice were divided into four groups: control, PSC-Fe_2_O_3_ alone, PSC-Fe_2_O_3_ plus mannitol intravenously, and PSC-Fe_2_O_3_ intravenously plus AA oral administration. The exposure dose and routine were chosen according to the results in vitro and the references [4, 32]. Results obtained from the immunofluorescence staining showed that the expression of CD31 was decreased and α-SMA increased in the liver, kidney and spleen of mice given PSC-Fe_2_O_3_ alone, while the addition of mannitol or AA rescued the level of the two markers, which clearly indicated that oxidative stress induced by PSC-Fe_2_O_3_ could induce EndMT in those organs, and the effect was rescued by the use of ROS scavengers (Fig. 7). The immunohistochemical staining with the liver tissue also provided side evidence of down regulation for CD31 and upregulation for α-SMA (Additional file 1: Figure S3), which was consistent with the result of immunofluorescence staining. As shown in previous reports, either human microvascular endothelial cell (HMVEC) or liver sinusoidal endothelial cells (LSEC) underwent leakiness through the disruption of VE-cad when exposed to titanium dioxide nanoparticles [33, 34], which suggested endothelial cells from different sources have some features in common.

**Fig. 8:**
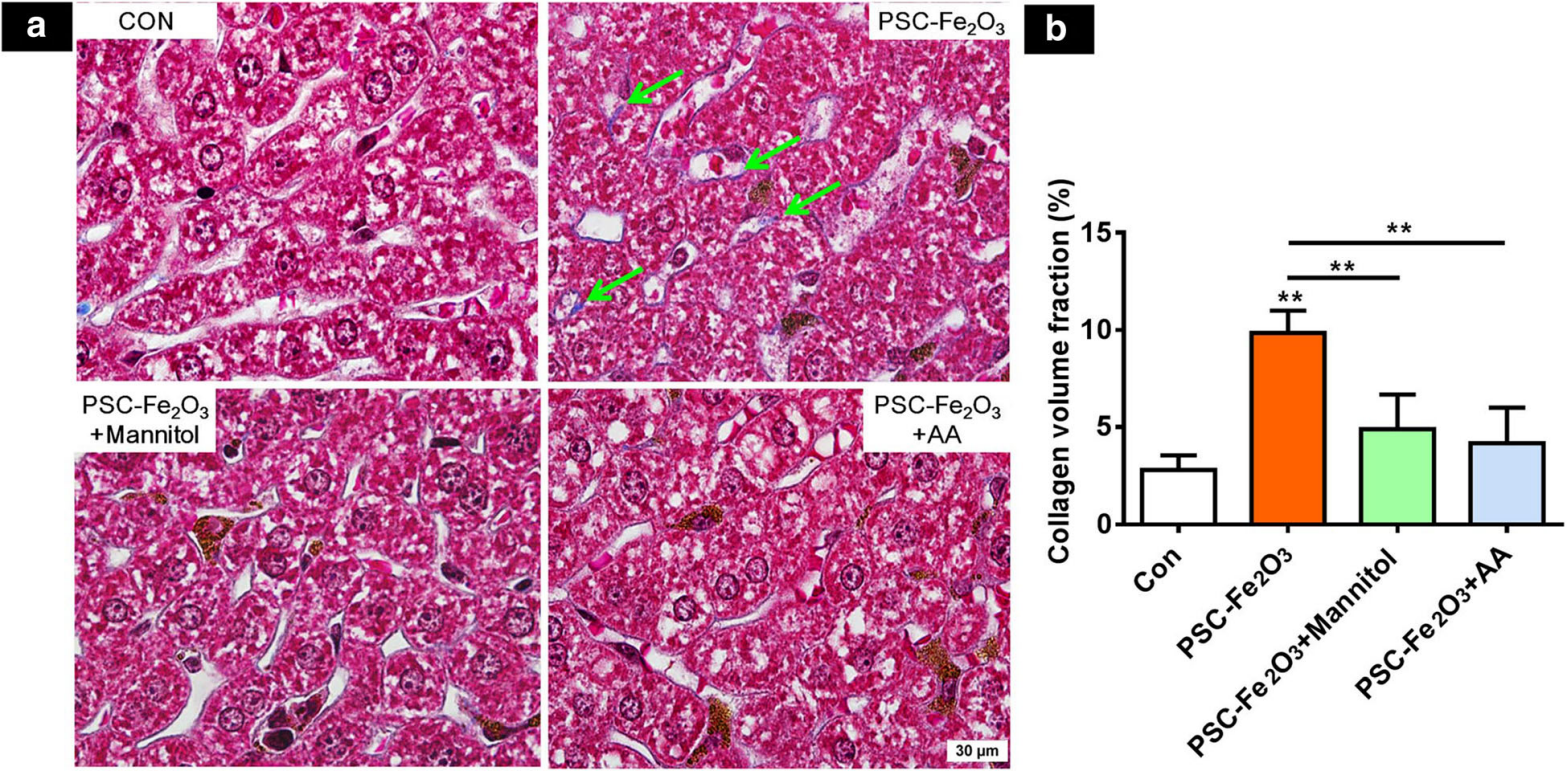
**a** Representative images of liver sections with Masson’s trichrome staining (blue color pointed by green arrows) for extracellular matrix collagen. **b** Quantification of collagen volume fraction. Data are mean ± SD, *n* = 4, one-way ANOVA with LSD-t, *p < 0.05, ** *p* < 0.01

In addition, results from Masson’s trichrome staining showed that the density of collagen fibers in the liver sinusoid of mice administrated with PSC-Fe_2_O_3_ alone was increased, which indicated the occurrence of liver fibrosis. The supplements of mannitol or AA could reduce the density of collagen fibers (Fig. 8).

## Discussion

Endothelial-to-mesenchymal transition (EndMT) is accompanied with a progressive loss of endothelial markers and cell-to-cell adhesion, transition towards a mesenchymal phenotype, and enhancement on cell migration and invasion, which has been recognized to play a diverse role in many diseases, such as fibrosis, systemic sclerosis, diabetes and malignancy [10]. In this work, we revealed that PSC-Fe_2_O_3_ could induce EndMT at acutely non-cytotoxic concentration. In order to figure out whether the occurrence of EndMT came from the modification of PSC, we also performed experiments with bare Fe_2_O_3_ nanoparticles (bare-Fe_2_O_3_) of 10 nm in diameter (Additional file 1: Fig. S4A). The bare-Fe_2_O_3_ showed 50% of viability when the concentration was reached 300 μg/mL (Additional file 1: Figure S4B), and induced significant morphological changes of HUVECs, cells shape changed from polygonal to spindle at different concentrations less than 300 μg/mL (Additional file 1: Figure S4C). Different from the cellular uptake of PSC-Fe_2_O_3_, the bare-Fe_2_O_3_ was able to be engulfed by HUVECs evidenced by the increasing blue color in the cells. In further, western blot analysis showed that α-SMA was increased remarkably and VE-cad decreased at the same time in dose-dependent manner (Additional file 1: Figure S4D). These results indicated that bare-Fe_2_O_3_ was capable of inducing EndMT on HUVECs as well, suggesting that the induction of EndMT was closely related with the intrinsic property of NPs instead of surface modification and cellular uptake. Note, both PSC-Fe_2_O_3_ and bare-Fe_2_O_3_ were household, which should not be taken as equal ones to commercial products in application, considering that they may have different physicochemical properties. It was known that EndMT is a subtype of epithelial-mesenchymal transition (EMT). Several nanoparticles or nanostructures have been demonstrated to be able to induce EMT with epithelial cell in the pulmonary system or cancer cell, such as carbon nanotube [35–38], cerium oxide [39], titanium dioxide (TiO_2_) [40], silica (SiO_2_) [41], silver NPs [42], which is supportive to our observations on PSC-Fe_2_O_3_-induced EndMT. These insights about PSC-Fe_2_O_3_-induced EndMT can also provide a new view to evaluate the potential health risk of this nanoparticle in further applications.

IONPs are able to induce the production of intracellular ROS because they are internalized by cells and located in the lysosomes where the microenvironment is acidic [43]. Due to the surface modification, PSC-Fe_2_O_3_ were not engulfed by HUVECs, instead, they mainly adhered to the cell membrane or stayed in the intercellular space. Nevertheless, PSC-Fe_2_O_3_ were still capable of up-regulating the level of intracellular ROS by catalyzing extracellular H_2_O_2_ into ·OH that further induced oxidative stress of the cells and affected intracellular environment. Therefore we suggested that the intrinsic peroxidase-like activity of PSC-Fe_2_O_3_ brought out the EndMT effect. According to the mechanism, it was reasonable to consider that ROS scavengers could reverse the effect, which was validated by this study in vitro and in vivo. It was also important and encouraging to see that the supplementary of mannitol or AA could reduce the degree of EndMT, which implied that the combination of antioxidants with PSC-Fe_2_O_3_ was a promising way of prevention the risk of EndMT related toxicology for the pre- and clinical application with PSC-Fe_2_O_3_. Moreover, it was noticed that iron oxide nanoparticles could reduce the adherens conjugation between HUVECs and led to endothelial leakiness through increasing the production of ROS [21], which might be one of the potential mechanisms that trigger the occurrence of EndMT mediated by PSC-Fe_2_O_3_.

## Conclusion

The peroxidase-like activity of PSC-Fe_2_O_3_ resulted in the up-regulation of intracellular ROS though they were mainly stay in the culture medium and intercellular space, which induced EndMT on HUVECs with the decreased level of CD31 and VE-cadherin, and increased expression of α-SMA and FSP. ROS scavenger mannitol or AA could rescue the EndMT induced by PSC-Fe_2_O_3_ in vitro and in vivo. These results provided a new insight into the potential toxicity of PSC-Fe_2_O_3_ on vascular endothelial cells and demonstrated ROS scavenge was an effective strategy to reverse the process and reduce the potential toxicity.

## Methods

### PSC-Fe2O3 synthesis and characterization

The polydextrose sorbitol carboxymethyl ether (PSC) coated γ-Fe_2_O_3_ nanoparticles (PSC-Fe_2_O_3_) were synthetized by alternating-current magnetic field inducing method [44]. Bare Fe_2_O_3_ nanoparticles were prepared similar to PSC-Fe_2_O_3_ in the absent of PSC. The morphology of Fe_2_O_3_ was observed by transmission electron microscope (TEM-1400plus, JEOL). The hydrodynamic diameters and Zeta potential of PSC-Fe2O3 were detected by a Zetasizer Nano ZS90 analyzer (Malvern). Each measurement was repeated four times.

### Cell culture

Human umbilical vein endothelial cells (HUVECs, #8000), endothelial cell medium (ECM, #1001), fetal bovine serum (FBS, #0025), penicillin/streptomycin solution (P/S, #0503), endothelial cell growth supplement (ECGS,#1052) and Poly-L-Lysine (PLL, #0403) were all purchased from ScienCell Research Laboratories (San Diego, CA). HUVECs were grown on PLL coated culture plate in ECM supplemented with 5% FBS, 1% P/S and 1% ECGS.

### Cell viability assay

The viability of HUVECs incubated with Fe2O3 was analyzed using a cell count kit (CCK-8, Dojindo). The Fe2O3 was diluted in fresh medium to yield a final iron concentration that ranged from 0 to 600 μg/mL. After 24 h or 48 h incubation, the cells were rinsed twice with PBS and incubated with medium containing 10 μL CCK-8 reagents for 2 h. The absorbance of the medium was measured at 450 nm using a microplate reader (BioTek Synergy4). All measurements were carried out in triplicate, and the cell viability was calculated with the protocol provided by the manufacture.

### Cell morphology assay

HUVEC cells were exposed to 0, 300 and 600 μg/mL PSC-Fe_2_O_3_. After 48 h, the morphology of cells was observed by the microscope (Olympus IX71). The length and diameter of thirty cells in each treatment was measured by ImageJ software, and the ratio of length to diameter (L/D) was calculated.

### Immunoblotting

Cells were incubated with 0, 150, 300 and 600 μg/mL Fe_2_O_3_ for 48 h at 37 °C. HUVECs were lysed in RIPA buffer and analyzed by western blot. Primary antibodies were as follows: anti-VE-cadherin (#2500S, Cell Signaling Technology, Danvers, MA), anti-CD31 (#3528, CST), anti-α-smooth muscle actin (#19245S, CST), anti-FSP (#13018, CST) and anti-GAPDH (sc-25778, Santa Cruz). Protein bands were quantified using ImageJ software. For the rescue experiment, HUVECs were co-cultured by Fe_2_O_3_ with 0.88 mg/mL mannitol or 8.8 mg/mL ascorbic acid (AA).

### Immunofluorescent staining

Cells were treated with different concentrations of PSC-Fe_2_O_3_ for 48 h and then fixed with 4% paraformaldehyde for 15 min. After rinsing with PBS, cells were incubated with blocking solution (1% bovine serum albumin (BSA) in PBS with 0.3% Triton) for 1 h at room temperature. The following primary antibodies were applied overnight at 4 °C: anti-VE-cadherin, anti-CD31, anti-α-smooth muscle actin, and anti-FSP. Cells were washed with PBS, and corresponding fluorescent secondary antibodies (CST) were applied for 1 h at room temperature. Nuclear staining was performed use 4′,6-diamidino-2-phenylindole (DAPI, Zhongshan Goldenbridge). Micrographs were taken by the FluoView FV1000 confocal microscope (Olympus), and analyzed with ImageJ software. For the rescue experiment, the cells were treated with 600 μg/mL PSC-Fe_2_O_3_ and 0.88 mg/mL mannitol or 8.8 mg/mL AA. The paraffin-embedded liver, spleen, and renal sections were deparaffinized with xylene. After treatment with 3% BSA for 30 min, sections were incubated overnight with primary CD31 and α-SMA antibody. Slides were then washed and incubated in the dark for 50 min with corresponding alexa fluor® 488 or CY3 labeled secondary antibody. Slides were then again washed in PBS and counterstained with DAPI-containing mounting medium.

### Tubule formation

Cells were pretreated with 600 μg/mL PSC-Fe_2_O_3_ in the absent or present of 50 mM AA for 48 h before seeded in matrigel coated 96-well plates. Cells were allowed to attach for 8 h, stained with Calcien AM (Molecular Probe) and photographed by the fluorescence microscope (Olympus, IX71). Numbers of tubules were quantified using ImageJ with the Angiogenesis Analyzer plugin. Data presented were from three independent experiments.

### Wound-healing assay

Cells were pretreated with 600 μg/mL PSC-Fe_2_O_3_ with or without 50 mM AA for 48 h and then starved without FBS for 12 h. Wounds were made with 200 μL sterile pipette tip and washed with PBS to eliminate non adherent cells, thus a wound approximate 165 μm was created. Then cells were cultured with medium with 0.5% FBS and images were acquired at 0 and 12 h (Olympus IX71). Movements of cells were calculated using ImageJ software. The changes of scratch width were calculated with initial wound width subtracted to that of 12 h and then divided by the initial one.

### Prussian blue staining

Cells were treated with different concentrations of Fe_2_O_3_ for 48 h. After removing the culture medium, the cells were washed with PBS for three times and fixed with 4% paraformaldehyde for 30 min. The fixed cells were incubated with Prussian blue solution containing 5% hydrochloric acid aqueous solution and 5% potassium ferrocyanide (II) trihydrate for 30 min to determine the intracellular iron followed by staining with nuclear fast red. The cells were placed on an inverted microscope for observation.

### Transmission electron microscopy (TEM)

Cells were treated with 600 μg/mL PSC-Fe_2_O_3_ for 48 h and then detached with trypsin and fixed overnight with 2.5% glutaraldehyde at 4 °C. The samples were then postfixed in Osmium (VIII) tetroxide (OsO_4_), dehydrated in ethanol, and embedded in Epon (Fluka). Sections were cut with an ultra-microtome and placed on copper grids for observation (TEM-1400 plus, JEOL).

### Iron content measurements

Cells were treated with PSC-Fe_2_O_3_ for 48 h. There are two different procedures to deal with HUVECs before detecting the iron content in cells. Procedure I: the cells were washed with PBS and lysed by 100 μL RIPA. Procedure II: the cells washed with PBS were detached from the plates with trypsin and collected by centrifugation, and then washed with PBS and lysed by 100 μL RIPA. The amount of iron was quantified with phenanthroline spectrophotometric method [45]. The iron content in the culture medium was determined with the same method. Standard calibration of iron amount and absorbance at 510 nm was built by using series concentration (0, 20, 40, 60, 80, 100 μg/mL) of FeCl_3_ solution. The concentrations of iron in the lysates and medium were calculated from the standard curve. Data presented were from three independent experiments performed in triplicate.

### Intracellular reactive oxygen species (ROS) measurement

The intracellular ROS level was measured using the probe 2′,7′-dichlorodihydrofluorescein diacetate (DCFH-DA, Sigma-Aldrich). HUVECs were treated with various concentration of PSC-Fe_2_O_3_ for 24 h and the culture supernatant was collected. The cells were then washed with PBS for three times. Cells were incubated with 10 μM DCFH-DA for 30 min and then digested and collected. The fluorescence intensity was detected by flow cytometer (BD Accuri C6). For the rescue experiment, the cells were treated by PSC-Fe_2_O_3_ with 0.88 mg/mL mannitol or 8.8 mg/mL AA. Data presented were from three independent experiments performed in triplicate.

### Extracellular H_2_O_2_ and ROS measurement

The extracellular H_2_O_2_ and ROS level was measured using the above collected supernatants. In order to avoid interference from PSC-Fe_2_O_3_, ultrafiltration centrifugal filter (10 K, Amicon®) was used to separate PSC-Fe_2_O_3_ from supernatants. Centrifugal filter was performed following the manufacturer’s recommendations. In short, the supernatants were added to the device, followed by centrifugation. Then, the solution in the filter collection tube was collected.

The H_2_O_2_ concentration in the filtered solution was detected by hydrogen peroxide assay Kit (Beyotime Biotechnology). Standard calibration of H_2_O_2_ was built by using series concentration (1, 2, 5, 10, 20 μM) of H_2_O_2_ solution (Beijing Chemical Works) with absorbance at 560 nm. The concentration of H_2_O_2_ in the filtered solution was calculated with the standard curve. Data from three independent experiments were presented.

To explore the effect of PSC-Fe_2_O_3_ on extracellular ROS production, we used a modified assay with DCFH-DA probe [46]. DCFH-DA in DMSO was hydrolyzed in NaOH aqueous solution for 30 min to yield DCFH intermediate. The obtained DCFH solution was neutralized with NaH_2_PO_4_ and shielded from light. The obtained filtered supernatants were mixed with DCFH solution. After 30-min incubation, the fluorescence was detected (BioTek Synergy4) and the excitation and emission wavelengths were set at 488 and 525 nm, respectively.

### qPCR

Cells were incubated with 0, 150, 300 and 600 μg/mL PSC-Fe_2_O_3_ for 24 h at 37 °C. The total RNA of the cells was extracted by using trizol (MKCB2851V, Sigma) with one-step extraction. Total RNA was transcribed using the cDNA synthesis kit (#RR036A, TaKaRa). Quantitative real-time-PCR (qPCR) was performed using the real-time PCR system (Bio-Rad) and TB Green fluorescein mix (#RR420A, TaKaRa). Data were normalized based on GAPDH expression as housekeeping gene. All primers were synthesized from TSINGKE Biological Technology and shown in Table 1.

**Table 1:** Sequences information of the gene primers

	Forward	Reverse
*HO-1*	AAGACTGCGTTCCTGCTCAAC	AAAGCCCTACAGCAACTGTCG
GAPDH	GTCAAGCTCATTTCCTGGT	CCAGGGTTTCTTACTCCTTG

### In vitro peroxidase-like activity assay

The peroxidase-like activity of PSC-Fe_2_O_3_ was evaluated in deionized water with the catalytic oxidation of o-phenylenediamine (OPD). The peroxidase-like activities of different concentrations (0, 25, 50, 60 μg/mL) PSC-Fe_2_O_3_ were employed with 45 μg/mL OPD and 370 mM H_2_O_2_. The progress was monitored with UV-vis-NIR spectrophotometer (PerkinElmer Lambda 950) with 1-min intervals at 440 nm. All operations were done at room temperature and in the dark.

### Electron spin resonance (ESR)

Hydroxyl radicals (·OH) can be detected by ESR with the help of the spin trap 5,5-Dimethyl-1-pyrroline N-oxide (DMPO, Dojindo). The ESR measurements were carried out using a Magnettech ESR spectrometer (MS-5000) at ambient temperature. 50 μL aliquots of control or sample solutions were taken in quartz capillary tubes. Other settings were as follows: 2 G field modulation, 100 G scan range, and 10 mW microwave power.

### Animal experiments

Animal experiments were carried out in accordance with a protocol that was approved by the Institutional Animal Care and Use committee (Institute of Basic Medical Sciences, Chinese Academy of Medical Sciences, and Peking Union Medical College). Female Balb/c mice (average weight 20 g) were maintained at the institutional experimental animal center under specific pathogen-free conditions. The mice were fed with sterilized food and autoclaved tap water.

Mice were divided into four groups, and each group consisted of three mice. Mice were intravenous injection of PSC-Fe_2_O_3_ or in combination with mannitol or oral administration AA as the following table showed (Table 2).

**Table 2:** The administration procedure of mice

Group	Intragastrical administration (100 μL)	intravenous injection (100 μL)
Control	saline	saline
PSC-Fe_2_O_3_	saline	4 mg/mL PSC-Fe_2_O_3_
PSC-Fe_2_O_3_ + AA	40 mg/mL AA	4 mg/mL PSC-Fe_2_O_3_
PSC-Fe_2_O_3_ + mannitol	saline	4 mg/mL PSC-Fe_2_O_3_ + 5.87 mg/mL mannitol

Note: saline was 0.9% sodium chloride

The mice were treated once a day for 7 days and then sacrificed. The tissues were collected and fixed in 4% parformaldehyde for 24 h and subsequently embedded in paraffin for histopathological and immunohistochemistry analysis.

### Histopathological and immunohistochemistry analysis

The paraffin-embedded tissue sections were deparaffinized with xylene. Endogenous peroxidase was blocked with 0.3% H_2_O_2_ for 25 min. After treatment with 3% BSA for 30 min, sections were incubated overnight with primary CD31 and α-SMA antibody. Slides were then washed and incubated with a horse radish peroxidase-linked secondary antibody for 50 min at room temperature, followed by diaminobenzidine and counterstaining with Mayer’s hematoxylin.

The slides were stained with Masson’s trichrome for evaluation of interstitial collagen deposition. Collagen area and total tissue area were measured using ImageJ and the IHC Tool Box plugin. Collagen volume fraction (CVF) was calculated dividing collagen area by the total area. For each group, four replicates were counted and calculated.

### Statistical analysis

The data are shown as mean ± standard deviation (SD) for all treatment groups. Statistical significance was ascertained through one way ANOVA with SPSS software (SPSS17.0).

## Additional file

Additional file 1: **Figure S1.** Representative morphology of low density of HUVECs treated with 300 and 600 μg/mL PSC-Fe_2_O_3_. **Figure S2.** PSC-Fe_2_O_3_ inhibited CD31 protein and gene expression, which was rescued by antioxidants. (A) Three replications of CD31 immunoblots. (B) Quantification of CD31 expression for HUVECs with different treatments. (C) Gene expression of CD31 for endothelial cell with different treatments. Data are mean ± SD, one-way ANOVA with LSD-t, ** *p* < 0.01. **Figure S3.** Representative of liver sections stained for mesenchymal markers α-SMA, and the endothelial markers CD31. **Figure S4.** Effects of bare Fe_2_O_3_ nanoparticles (bare-Fe_2_O_3_) on HUVECs. (A) TEM image for bare-Fe_2_O_3_. (B) Cell viability of HUVECs treated with different concentrations of bare-Fe_2_O_3_ for 24 h or 48 h. (C) Representative immunoblots of VE-Cadherin and α-SMA of HUVECs treated with 0, 10, 50 and 300 μg/mL of bare-Fe_2_O_3_ for 48 h. (D) Microscope observation of HUVEC cells treated with different concentrations of bare-Fe_2_O_3_ after Prussian blue staining. (DOCX 1010 kb)

## Data Availability

The datasets used and/or analysed during the current study are available from the corresponding author on reasonable request.

## Abbreviations

AA: ascorbic acid
DCFH-DA: 2′, 7′-dichlorodihydrofluorescein diacetate
DMPO: 5, 5-Dimethyl-1-pyrroline N-oxide
ECs: Endothelial cell
EndMT: Endothelial-to-mesenchymal transition
ESR: Electron spin resonance
FSP: Fibroblast specific protein
HUVEC: Human umbilical vein endothelial cells
IONPs: Iron oxide nanoparticles
OH: Hydroxyl radicals
OPD: o-phenylenediamine
PSC-Fe_2_O_3_: γ-Fe_2_O_3_ nanoparticle core and polyglucose sorbitol carboxymethyether shell
ROS: Reactive oxygen species
TEM: Transmission electron microscope
α-SMA: α-smooth muscle actin
