# Supplementary material for: Iron oxide nanoparticles induce reversible endothelial-to-mesenchymal transition in vascular endothelial cells at acutely non-cytotoxic concentrations
Source: Part Fibre Toxicol. 2019 Jul 12;16:30. doi: 10.1186/s12989-019-0314-4 (PMC6626375; doi:10.1186/s12989-019-0314-4)
Supplement: Supplementary file 1 — Figure S1. Representative morphology of low density of HUVECs treated with 300 and 600 μg/mL PSC-Fe2O3. Figure S2. PSC-Fe2O3 inhibited CD31 protein and gene expression, which was rescued by antioxidants. (A) Three replications of CD31 immunoblots. (B) Quantification of CD31 expression for HUVECs with different treatments. (C) Gene expression of CD31 for endothelial cell with different treatments. Data are mean ± SD, one-way ANOVA with LSD-t, ** p < 0.01. Figure S3. Representative of liver sections stained for mesenchymal markers α-SMA, and the endothelial markers CD31. Figure S4. Effects of bare Fe2O3 nanoparticles (bare-Fe2O3) on HUVECs. (A) TEM image for bare-Fe2O3. (B) Cell viability of HUVECs treated with different concentrations of bare-Fe2O3 for 24 h or 48 h. (C) Representative immunoblots of VE-Cadherin and α-SMA of HUVECs treated with 0, 10, 50 and 300 μg/mL of bare-Fe2O3 for 48 h. (D) Microscope observation of HUVEC cells treated with different concentrations of bare-Fe2O3 after Prussian blue staining. (DOCX 1010 kb) [file 12989_2019_314_MOESM1_ESM.docx]

**Supporting information**

**Iron oxide nanoparticles induce reversible endothelial-to-mesenchymal transition in vascular endothelial cells at acutely non-cytotoxic concentrations**

Tao Wen^1#^, Lifan Du^1#^, Bo Chen^2^, Doudou Yan^1^, Aiyun Yang^1^, Jian Liu^1^, Ning Gu^3^, Jie Meng^1*^, Haiyan Xu^1*^

1. Institute of Basic Medical Sciences, Chinese Academy of Medical Sciences & Peking Union Medical College, Beijing 100005, China
2. Materials Science and Devices Institute, Suzhou University of science and technology, Suzhou, 215009, China
3. State Key Laboratory of Bioelectronics, Jiangsu Key Laboratory for Biomaterials and Devices, School of Biological Science and Medical Engineering, Southeast University, Nanjing 210096, China


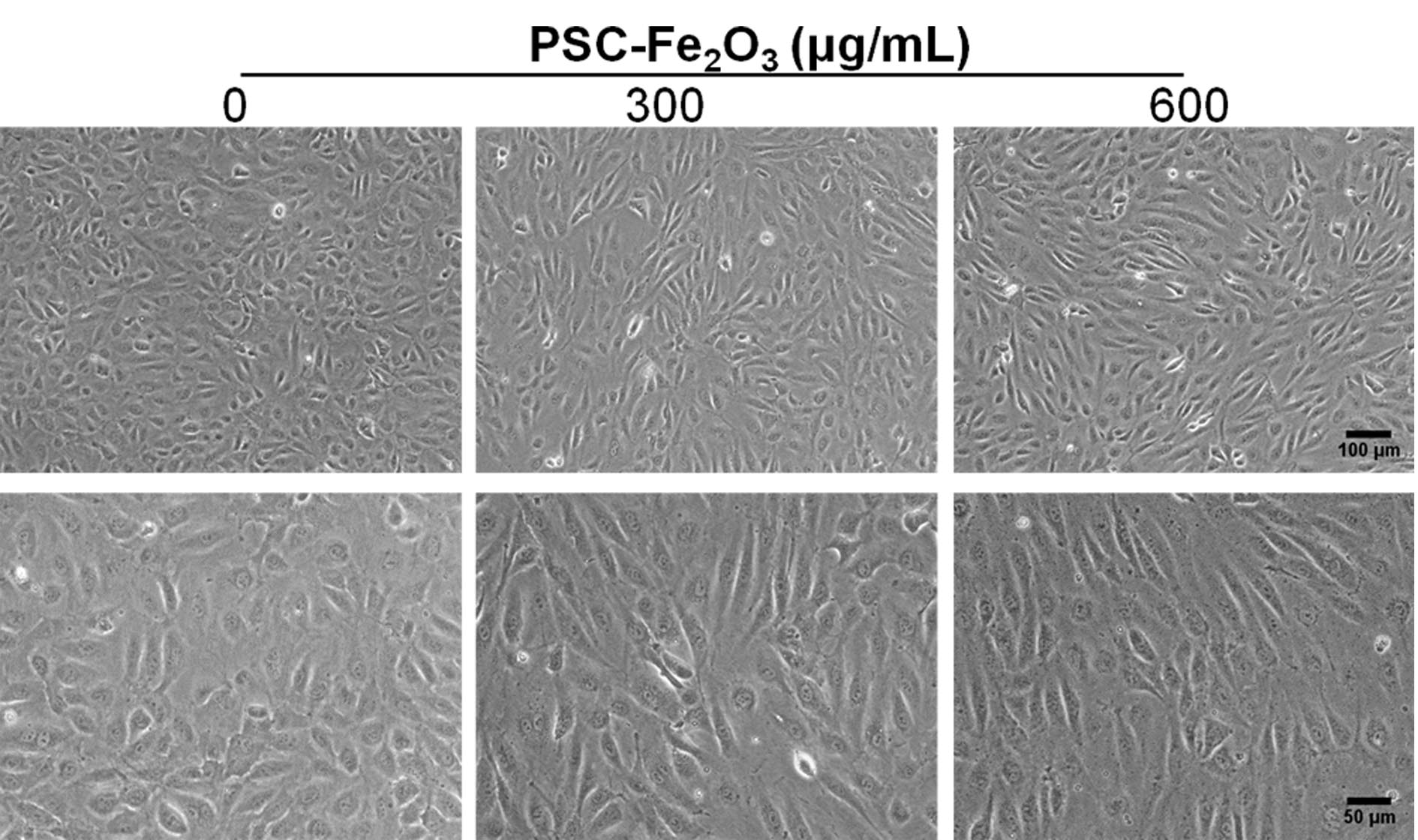


**Figure S1** Representative morphology of low density of HUVECs treated with 300 and 600 μg/mL PSC-Fe_2_O_3_.


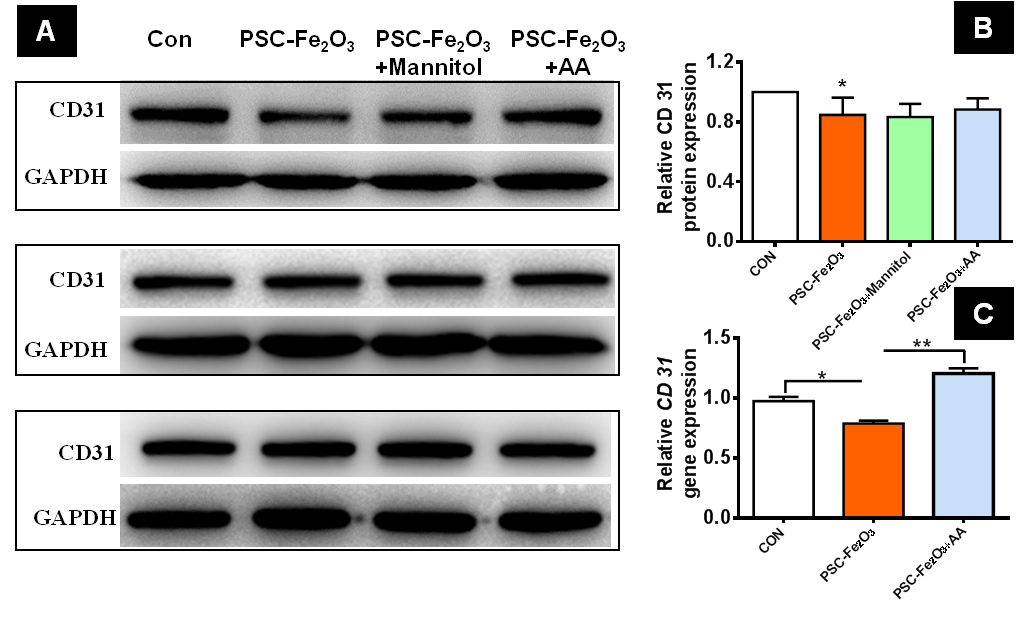


**Figure S2** PSC-Fe_2_O_3_ inhibited CD31 protein and gene expression, which was rescued by antioxidants. (A) Three replications of CD31 immunoblots. (B) Quantification of CD31 expression for HUVECs with different treatments. (C) Gene expression of CD31 for endothelial cell with different treatments. Data are mean ± SD, one-way ANOVA with LSD-t, ** p < 0.01.


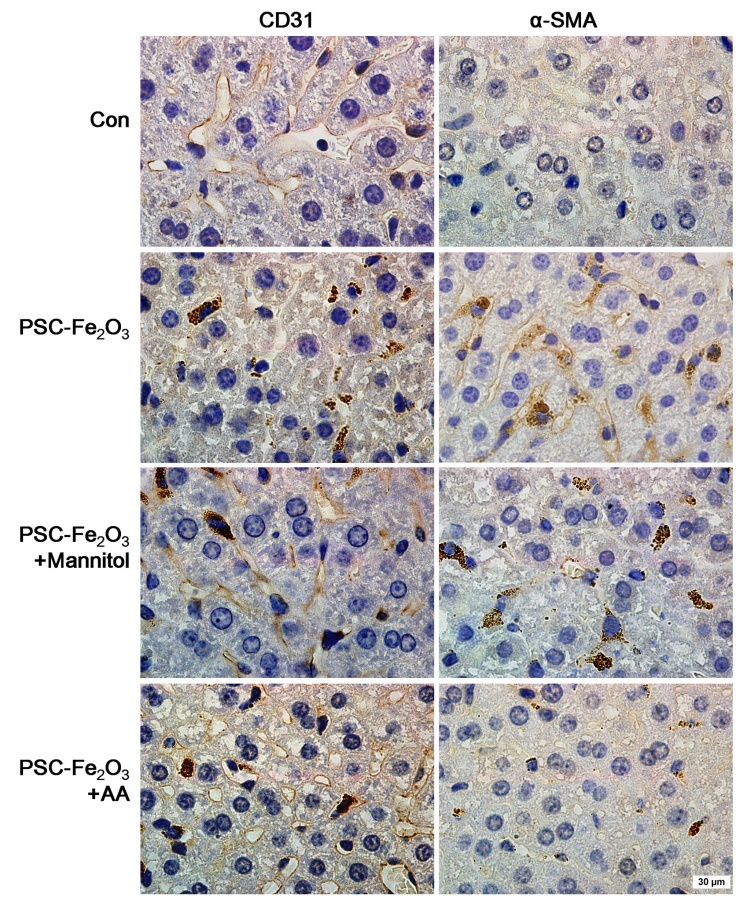


**Figure S3**. Representative of liver sections stained for mesenchymal markers α-SMA, and the endothelial markers CD31.


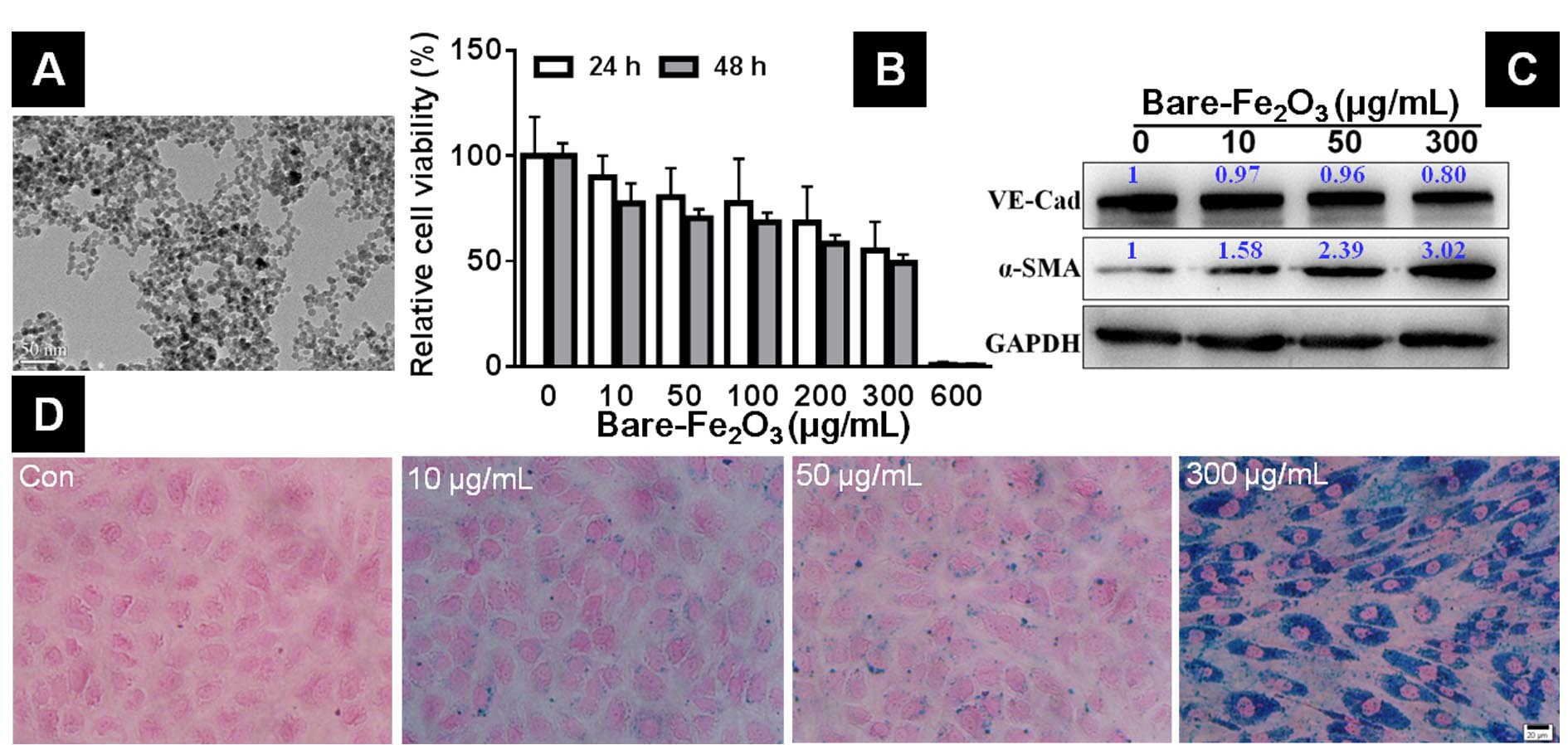


**Figure S4** Effects of bare Fe_2_O_3_ nanoparticles (bare-Fe_2_O_3_) on HUVECs. (A) TEM image for bare-Fe_2_O_3_. (B) Cell viability of HUVECs treated with different concentrations of bare-Fe_2_O_3_ for 24 h or 48 h. (C) Representative immunoblots of VE-Cadherin and α-SMA of HUVECs treated with 0, 10, 50 and 300 μg/mL of bare-Fe_2_O_3_ for 48 h. (D) Microscope observation of HUVEC cells treated with different concentrations of bare-Fe_2_O_3_ after Prussian blue staining.
